# Is Vitamin D Insufficiency Associated with Mortality of Critically Ill Patients?

**DOI:** 10.1155/2013/856747

**Published:** 2013-12-25

**Authors:** Gulbin Aygencel, Melda Turkoglu, Ayse Fitnat Tuncel, Burcu Arslan Candır, Yelda Deligoz Bildacı, Hatice Pasaoglu

**Affiliations:** ^1^Division of Critical Care Medicine, Department of Internal Medicine, Gazi University Faculty of Medicine, Besevler, 06510 Ankara, Turkey; ^2^Department of Medical Biochemistry, Gazi University Faculty of Medicine, Besevler, 06510 Ankara, Turkey

## Abstract

*Objective*. To evaluate the vitamin D status of our critically ill patients and its relevance to mortality. *Patients and Methods*. We performed a prospective observational study in the medical intensive care unit of a university hospital between October 2009 and March 2011. Vitamin D levels were measured and insufficiency was defined as <20 ng/mL. *Results*. Two hundred and one patients were included in the study. The median age was 66 (56–77) and the majority of patients were male (56%). The median serum level of vitamin D was 14,9 ng/mL and 139 (69%) patients were vitamin D insufficient on admission. While we grouped the ICU patients as vitamin D insufficient and sufficient, vitamin D insufficient patients had more severe acute diseases and worse laboratory values on admission. These patients had more morbidities and were exposed to more invasive therapies during stay. The mortality rate was significantly higher in the vitamin D insufficient group compared to the vitamin D sufficient group (43% versus 26%, *P* = 0,027). However, logistic regression analysis demonstrated that vitamin D insufficiency was not an independent risk factor for mortality. *Conclusion*. Vitamin D insufficiency is common in our critically ill patients (69%), but it is not an independent risk factor for mortality.

## 1. Introduction

Although vitamin D (vit D) is classified as a fat-soluble vitamin that plays an important role in bone metabolism, it is also a steroid hormone with pleiotropic effects. With its pleiotropic effects, vit D regulates immunity, inflammation, cell proliferation, differentiation, apoptosis, and angiogenesis. Several recent studies demonstrate a close relationship between vit D insufficiency and various systemic disorders associated with significant morbidity and mortality [1, 2].

Vitamin D insufficiency is a common global phenomenon. Its insufficiency is also reported to be common in hospitalized patients and particularly in critically ill patients [3, 4]. The incidence of vit D insufficiency in critically ill patients has been reported to range from 26% to 82% [5, 6]. This insufficiency may worsen existing immune and metabolic dysfunctions in critically ill patients, leading to worse outcomes [6, 7].

The purpose of this study was to evaluate vit D status in our critically ill patients and to demonstrate the relationship between vit D status and mortality.

## 2. Material and Method

We performed a prospective observational study in a 9-bed medical ICU at Gazi University Hospital, Ankara (39.875°N 32.833°E, mean elevation of 938 metres-3077ft-), Turkey. The medical ICU with an annual occupancy of 400 patients serves as a tertiary reference center for medical patients. The study has been approved by the Institutional Ethical Committee.

Patients consecutively admitted to the ICU between October 2009 and March 2011 were considered for the study. Patients who stayed in the ICU less than 48 hours, were aged <18 years, had terminal stage cancer, or were using drugs related to bone metabolism were excluded from the study. For readmitted patients, the first admission was recorded. Blood samples for vit D levels were drawn in the first 24 hours of ICU admission and were centrifuged at 3000 g for 15 minutes. Sera were stored at −80°C until analysis. Serum vit D (25(OH)D) levels were measured by radioimmunoassay (DIAsource, Belgium) and levels below 20 ng/mL were defined to be insufficient.

The following data was recorded and analyzed in the study: demographics; admission acute physiology and chronic health evaluation (APACHE) II score; sequential organ failure assessment (SOFA) score; Glasgow coma scale (GCS); underlying disease; cause of admission; admission biochemical data, various morbidities during ICU stay (mechanical ventilation, sepsis/septic shock, renal replacement therapy, nosocomial infections, etc.), and the length of ICU stay and mortality of patients.

### 2.1. Statistical Analysis

SPSS software (SPSS version 15.0) was used for statistical analysis. Continuous variables were summarized as medians [interquartile range]. Categorical variables were summarized as proportions (percentages). Comparisons between patients with vit D insufficiency and vit D sufficiency and survivor and nonsurvivor patients were performed using Pearson's chi-square or Fisher's exact test for categorical variables and the Mann-Whitney *U* test for continuous variables. We also analyzed the variables using quartiles of admission vit D levels. Comparisons of groups were performed using chi-square test for categorical variables and the Kruskal-Wallis *H* test for continuous variables. A multivariate analysis was performed for the whole study population, choosing ICU mortality as the dependent variable. Independent variables were the presence of vit D insufficiency, APACHE II score, procalcitonin and albumin level on admission, development of renal failure and sepsis/septic shock, applying of renal replacement therapy and mechanical ventilation, steroid use, invasive monitoring of blood pressure, and blood/blood products transfusion during ICU stay. A *P* value less than 0.05 was considered as statistically significant.

## 3. Results

**Figure 1:**
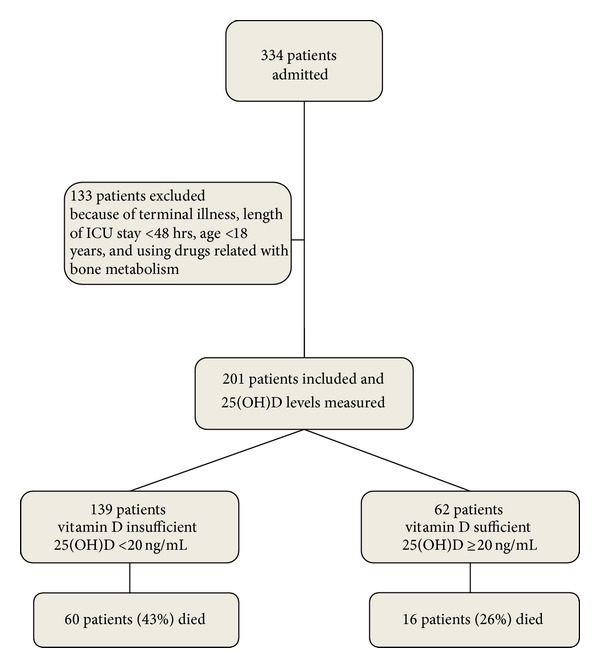
Study design.

Among 334 patients admitted to the ICU during the study period, 201 patients were included in the study (Figure 1). The median age was 66 [56–77] and the majority of patients were male (56%). The median serum level of 25(OH)D was 14,9 [7,5–26,4] ng/mL. Of 201 study patients, 139 (69%) were vitamin D insufficient.

The most common cause of admission to the ICU was respiratory insufficiency (45%). The most common comorbidity of ICU patients was cardiovascular disease (35%). The majority of patients were admitted to our medical ICU from emergency services (43%). On admission, two or more organ dysfunctions were found in 89 (44%) patients. Invasive mechanical ventilation was applied to 88 (44%) patients. Sepsis/septic shock was encountered in 72 (35,8%) patients. During ICU stay, most of the patients were monitored invasively. Renal replacement therapy was required in 69 (34%) patients, and nosocomial infection was detected in 71 (35%) patients. The most common developed infection was ventilator-associated pneumonia and the most common pathogen was *Acinetobacter* spp.  Table 1 shows some baseline characteristics of the study population.

**Figure 2:**
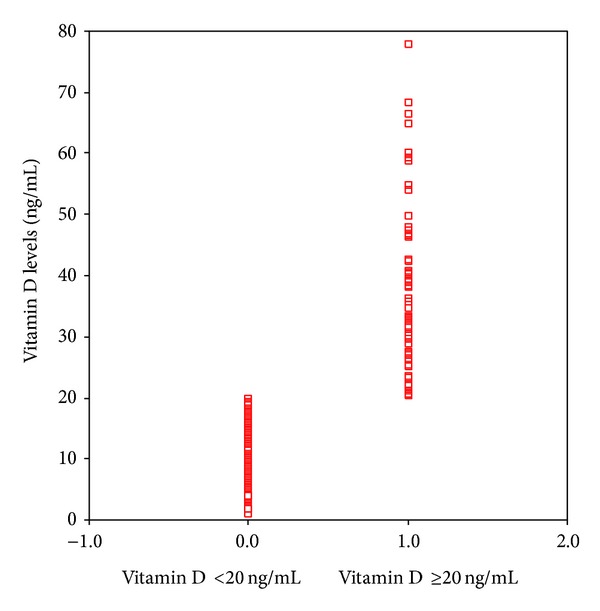
Vitamin D levels of vitamin D sufficient (≥20 ng/mL) and insufficient (<20 ng/mL) groups (*P* = 0.001).

**Figure 3:**
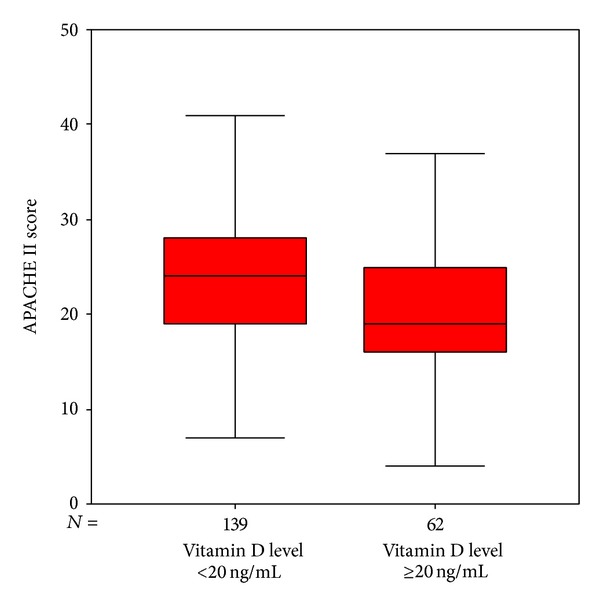
APACHE II scores of vitamin D sufficient and insufficient groups (*P* = 0.006).

**Figure 4:**
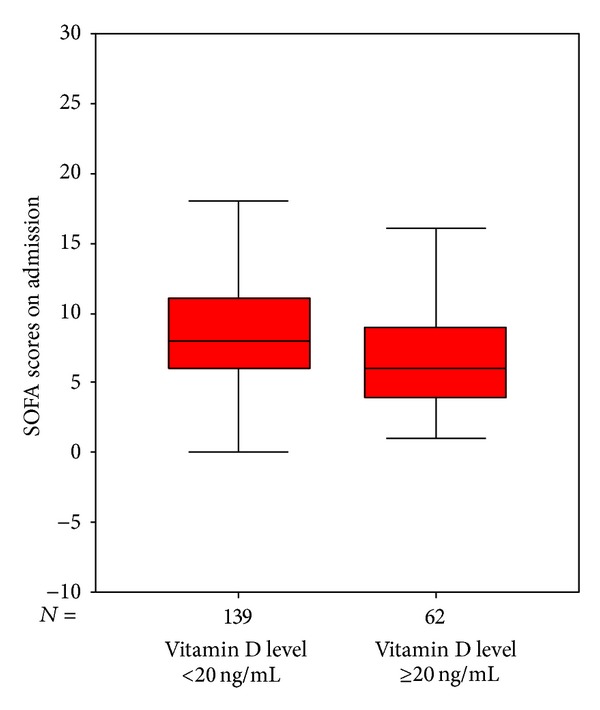
SOFA scores of vitamin D sufficient and insufficient groups (*P* = 0.005).

**Figure 5:**
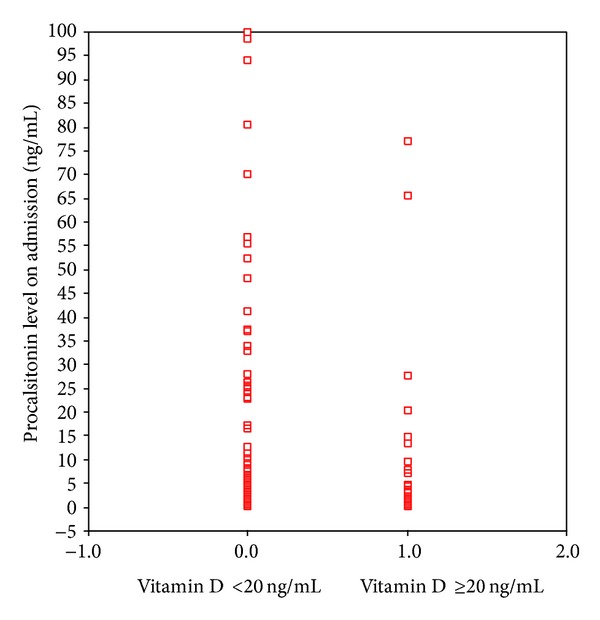
Procalsitonin levels of vitamin D sufficient and insufficient groups (*P* = 0.044).

**Figure 6:**
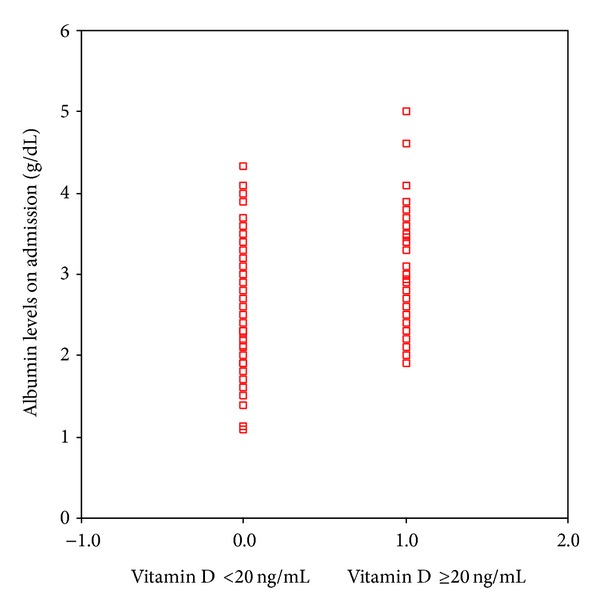
Albumin levels of vitamin D sufficient and insufficient groups (*P* = 0.01).

**Figure 7:**
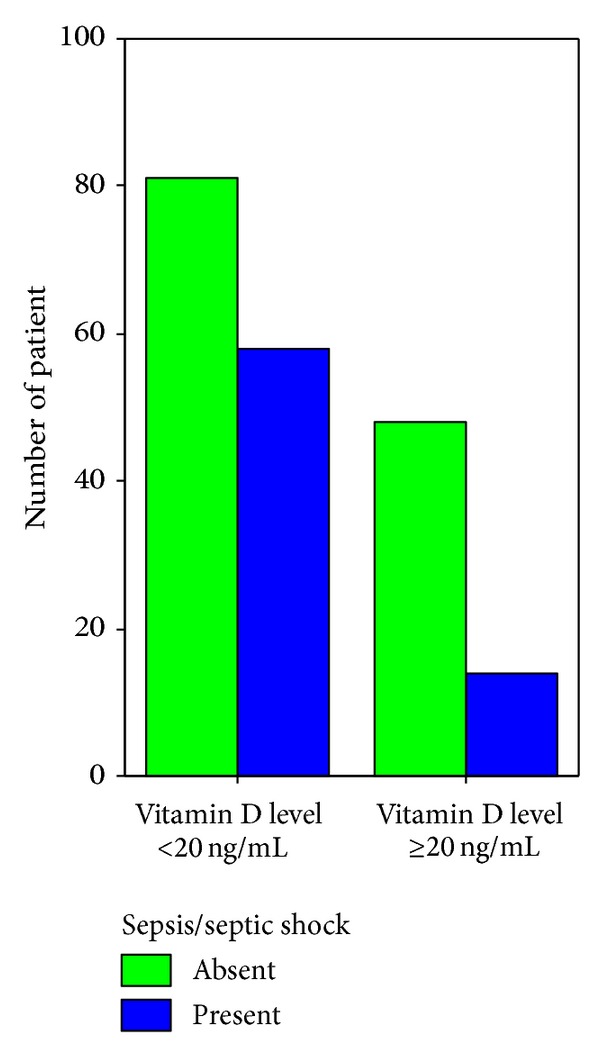
Sepsis/septic shock on admission in Vitamin D insufficient and sufficient patients (*P* = 0.026).

**Figure 8:**
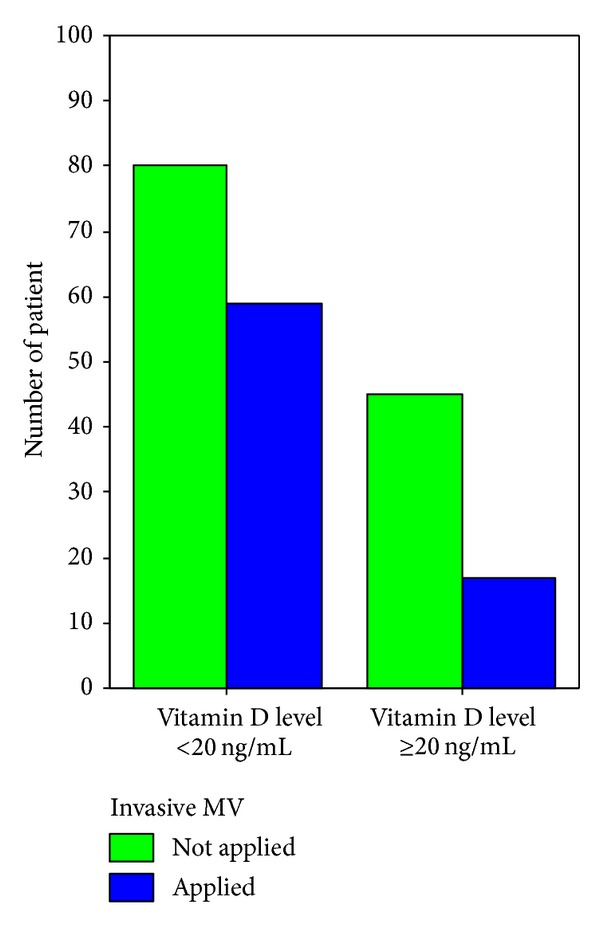
Applying invasive mechanical ventilation on admission in vitamin D insufficient and sufficient patients (*P* = 0.012).

**Figure 9:**
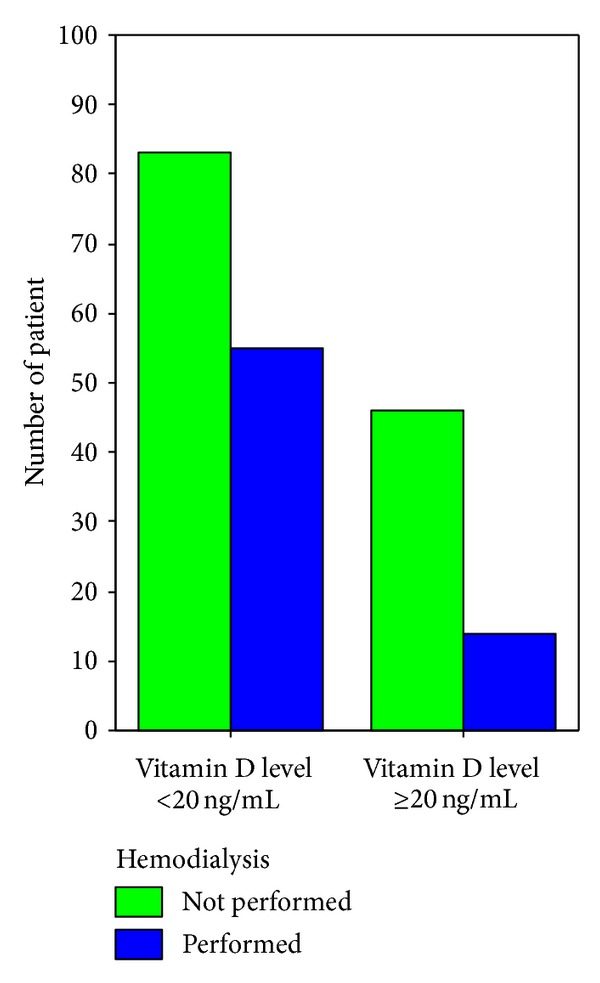
Performing hemodialysis during ICU stay in vitamin D insufficient and sufficient patients (*P* = 0.025).

**Figure 10:**
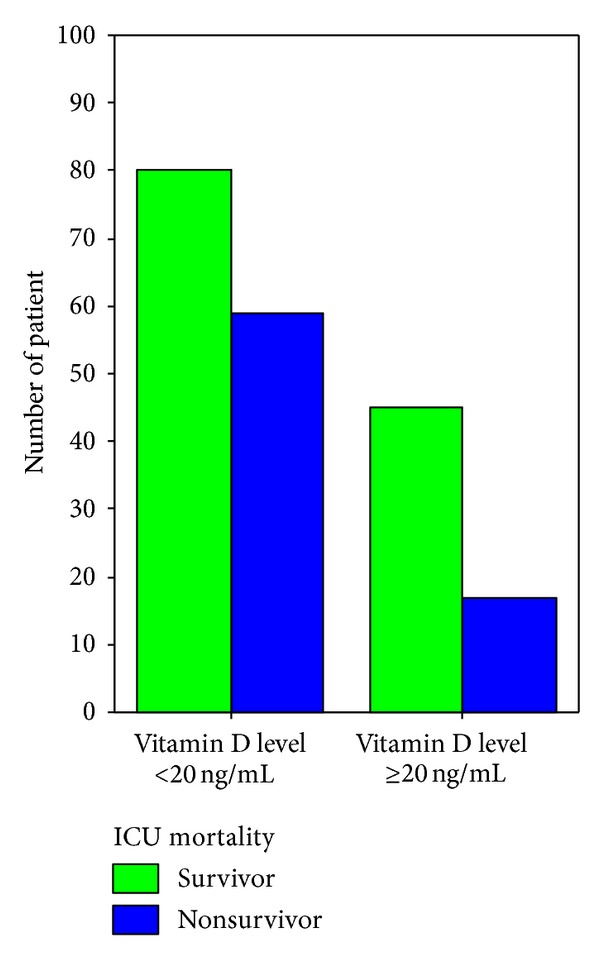
ICU mortality rate in vitamin D insufficient and sufficient patients (*P* = 0.027).

Patients were categorized in two groups in terms of their vit D status (vit D sufficient and vit D insufficient). It was evaluated whether a statistical difference existed between the groups. The median 25(OH) levels were 10 [6–16] ng/mL and 34 [28–46] ng/mL in the vit D insufficient and sufficient groups, respectively (*P* < 0.001). There were no significant differences between the two groups regarding demographics. Risk assessment scores including APACHE II and SOFA scores were higher in the vit D insufficient group. Leukocytes and procalsitonin levels on admission were significantly lower and ionized calcium, calcium, and albumin levels were significantly higher in the vit D sufficient group. Fewer cases of sepsis/septic shock on admission were found, fewer invasive mechanical ventilations were applied, and fewer organ dysfunctions were determined in the vit D sufficient group. During ICU stay, less invasive monitoring and fewer renal replacement therapies were performed in the vit D sufficient group. ICU mortality was significantly higher in the vit D insufficient group compared to the vit D sufficient group (see also Figures 2, 3, 4, 5, 6, 7, 8, 9, and 10).  Table 2 shows the differences between the vit D insufficient and sufficient groups.

We also separated the study group into four, using quartiles of admission vit D levels. Comparing these groups, we found significant differences in admission APACHE II and SOFA scores, procalsitonin, ionized calcium, albumin, and creatinine levels. Presence of sepsis/septic shock and renal dysfunction on admission and requirement of invasive mechanical ventilation and hemodialysis during ICU stay were also significantly different between the groups. But there were no differences in these groups according to ICU mortality (Table 3).

Patients who stayed in the ICU were grouped again into two: patients who survived (survivors discharge or transfer) (125 patients, 62%) and those who died (nonsurvivors) (76 patients, 38%). Significant differences were found between these groups when compared. The differences between survivor and nonsurvivors included risk assessment scores (APACHE II, SOFA, etc.), length of ICU stay, a number of laboratory values (hemoglobin, thrombocyte count, inflammatory markers, etc.), presence of vit D insufficiency/sufficiency, and various morbidities during ICU stay (nosocomial infections, renal replacement therapies, etc.).  Table 4 shows some characteristics of survivor and nonsurvivor patients in the ICU.

When logistic regression analysis was performed to identify factors independently associated with mortality in ICU, APACHE II score on admission, renal dysfunction, mechanical ventilation, presence of sepsis/septic shock, replacement of blood/blood products, and using steroids during ICU stay were significantly related with mortality (Table 5). However, vit D insufficiency was not an independent risk factor for mortality.

## 4. Discussion

Vitamin D sufficiency may be broadly defined as a circulating 25(OH)D level that satisfies physiologic needs. However, what level constitutes vit D sufficiency is extremely controversial. According to the 14th International Workshop Consensus Conference on Vitamin D, held in Belgium in October 2009, in the general population, a minimum 25(OH)D level of 20 ng/mL (50 nmol/L) is necessary to support bone and mineral health, and 30–40 ng/mL (75–100 nmol/L) is necessary to maintain muscle strength and immune functions [8]. Whether these definitions apply to critically ill patients is unclear. The most published studies to evaluate vit D insufficiency in critically ill patients have applied definitions used in the general population [4–6]. Therefore, we used a minimum 25(OH)D level (20 ng/mL) as a cutoff to define insufficiency in our ICU population.

Although different criteria for insufficiency were applied across the studies, reported prevalence of vit D insufficiency ranged from 26% to 82% in critically ill patients [5, 6]. This reported prevalence is close to 50% higher than that of patients in general medical wards [5, 6]. In our prospective observational study, we demonstrated that 69% of patients admitted to our medical ICU were vit D insufficient. The median 25(OH)D level was 14.9 [7.5–26.4] ng/mL.

Causes of low 25(OH)D levels in patients admitted to ICU are multifactorial. In addition to well-known etiologies (older age, sun avoidance, low dietary intake, comorbidities, etc.), it is important to consider other factors such as interaction with medications, abnormal gastrointestinal function and the effect of fluid resuscitation [9–11]. Very likely, 25(OH)D levels decline further during ICU stay because of insufficient replacement and absent sunlight exposure [5, 9–11]. We did not find this decline, as we did not sample 25(OH)D levels sequentially.

Most published studies show a higher prevalence of vit D insufficiency in women and the elderly in the general population [2]. We did not find any correlation between vit D insufficiency and age and gender in our critically ill patient population. Contrary to the study by McKinney et al., we did not find any correlation between 25(OH)D insufficiency and increased length of ICU stay (LOS) [10].

Vitamin D regulates both the innate and adaptive immune systems. Thus, vit D insufficiency leads to immune dysregulation. This immunopathy may manifest as increased susceptibility to nosocomial infections such as ventilator-associated pneumonia and increase the duration and severity of systemic inflammatory response and multiorgan failure [12–14]. In our study, we found a trend of increased nosocomial infections in vit D insufficiency (27% versus 39%), tending to confirm the suggestion of a causal relationship.

Standard vit D supplementation is incorporated in nutritional support in intensive care units but may be insufficient. Approximately 400–600 units of vit D are provided daily in standard enteral and parenteral nutritional support. Several studies demonstrate that current support is inadequate. High dose vit D supplementation may be more effective in critically ill patients. A number of recent studies in ICUs showed that high doses of vit D restored sufficiency within a short period (several days) without complications in critically ill patients [15]. Further research is warranted to determine the efficacy of vit D supplementation and the effective dose in critically ill patients.

Some studies suggest an association between vit D insufficiency and mortality in critically ill patients [16–19]. In the study by van den Berghe et al., vit D levels were lower among nonsurvivors in critically ill patients [20]. A study by Lee et al. revealed a threefold mortality rate in vit D insufficient patients compared to those who were sufficient (25(OH)D >24 ng/mL) [3]. A study by McKinney et al. revealed vit D insufficiency to be nearly twice as prevalent among nonsurvivors in ICU [10]. The increased mortality in critically ill patients with vit D insufficiency might be due to changes in glucose and calcium metabolism and/or immune and endothelial cell dysfunction [21, 22]. In our study, we found a correlation between mortality and vit D insufficiency in univariate analysis; however, vit D insufficiency was not found as an independent risk factor for mortality in multivariate analysis.

We believe that vit D insufficiency is a result of chronic, severe underlying diseases or comorbidities of a patient. Vitamin D insufficiency can be seen frequently in patients with chronic, severe diseases. They are also admitted to ICU with more severe acute illness, have worse severity scores and laboratory values, and also have a poor prognosis during ICU period and a high mortality rate. We also believe that vit D insufficiency is a helper but not a real risk factor for mortality.

Our study has several potential limitations. Firstly, it was a single center study with a small sample size; therefore, our study results may not be generalizable. Secondly, 25(OH)D levels obtained within 24 hours on admission are probably a reflection of preadmission insufficiency. We did not sample 25(OH)D levels sequentially and we did not follow up vit D levels during ICU stay. Finally, our study was conducted in a medical ICU and cannot be generalized to cardiac, surgical, or other ICUs.

In conclusion, vit D insufficiency is common in critically ill patients. Even though there was a statistical difference in mortality between vit D sufficient and insufficient patients, vit D insufficiency was not found to be an independent risk factor for mortality. Further multicenter studies with a larger sample size are required to validate our results.

## Figures and Tables

**Table 1:** Some baseline characteristics of study patients.

Variable	Study patients (*n* = 201)
Age (years)	66 [56–77]
Male gender	113 (56%)
Length of hospital stay prior to ICU admission (days)	4 [2–11]
Length of ICU stay (days)	9 [5–14]
On admission	
APACHE II score	23 [17–28]
Glasgow coma scale	14 [11–15]
SOFA score	7 [5–10]
Hemoglobin (g/dL)	10 [8.8–12]
Leukocytes (/mm^3^)	11800 [7200–17100]
Thrombocytes (/mm^3^)	149000 [90000–239000]
C-reactive protein (mg/L)	103 [36.8–180]
Procalsitonin (ng/mL)	1.9 [0.4–8.3]
Calcium level (mg/dL)	7.9 [7.2–8.6]
Calcium level, ionized (mmol/L)	1.15 [1.04–1.25]
Albumin level (g/dL)	2.7 [2.2–3.1]
Creatinine level (mg/dL)	1.6 [0.8–3.2]
Phosphorus level (mg/dL)	3.2 [2.2–4.6]
Oxygenation index (PaO_2_/FiO_2_)	188 [130–272]
25-hydroxyvitamin D (25(OH)D) level (ng/mL)	14.9 [7.5–26.4]
Development of nosocomial infection in ICU	71 (35%)
ICU mortality	76 (38%)

Values are presented as median [interquartile range] or *n* (%); APACHE: acute physiology and chronic health evaluation, SOFA: sequential organ failure assessment, and ICU: intensive care unit.

**Table 2:** Comparison of patients who were vitamin D insufficient and sufficient.

Variable	Vitamin D insufficient (<20 ng/mL) (*n* = 139)	Vitamin D sufficient (≥20 ng/mL) (*n* = 62)	*P* value
Age (years)	66 [58–78]	65 [42–77]	0.184
Male gender	72 (52%)	41 (66%)	0.06
Length of ICU stay (days)	9 [5–14]	8 [5–13]	0.355
On admission			
SOFA score	8 [6–11]	6 [4–9]	**0.005**
APACHE II score	24 [19–28]	19 [16–25]	**0.006**
25(OH)D level (ng/mL)	10 [6–16]	34 [28–46]	**0.001**
Leukocytes (/mm^3^)	12300 [8200–17500]	11150 [5370–14980]	**0.02**
Procalsitonin level (ng/mL)	3.7 [1.2–25.4]	1.5 [0.5–7.8]	**0.044**
Calcium (mg/dL)	7.8 [7.2–8.4]	8.2 [7.4–9]	**0.006**
Calcium, ionized (mmol/L)	1.12 [1–1.23]	1.22 [1.1–1.3]	**0.001**
Albumin (mg/dL)	2.6 [2.1–3.1]	2.8 [2.5–3.3]	**0.010**
Sepsis/septic shock	57 (41%)	15 (24.2%)	**0.026**
Invasive mechanical ventilation	69 (50%)	19 (31%)	**0.012**
Organ dysfunction	116 (84%)	44 (71%)	**0.042**
During ICU			
Invasive blood pressure monitoring	85 (64%)	26 (45%)	**0.014**
Central venous pressure monitoring	91 (69%)	28 (48%)	**0.007**
RRT (hemodialysis)	55 (40%)	14 (23%)	**0.025**
Nosocomial infections	54 (39%)	17 (27%)	0.117
ICU mortality	60 (43%)	16 (26%)	**0.027**

Values are presented as median [interquartile range] or *n* (%); APACHE: acute physiology and chronic health evaluation, SOFA: sequential organ failure assessment, and ICU: intensive care unit.

**Table 3:** Comparison of patients while separated four groups according to quartiles of admission vitamin D levels.

Variable	Group 1 (1st quartile) (*n* = 50)	Group 2 (2nd quartile) (*n* = 51)	Group 3 (3rd quartile) (*n* = 50)	Group 4 (4th quartile) (*n* = 50)	*P* value
25(OH)D level (ng/mL)	4.45 [3.13–6.19]	10.85 [9.04–13.22]	17.79 [16.44–19.92]	38.74 [31.65–47.47]	**0.0001**
Age (years)	65.5 [57.75–76]	71 [60–80]	65 [56.75–74]	62.5 [35.7–77]	0.115
Male gender	25 (50%)	25 (49%)	31 (62%)	32 (64%)	0.096
Length of hospital stay prior to ICU admission (days)	5 [2–14]	3 [2–6]	4 [2–15]	4 [1.75–11.5]	0.274
Length of ICU stay (days)	8,5 [5.75–20]	8 [5–11]	11 [6–15.25]	6 [4–12.5]	0,124
On admission					
APACHE II score	25.5 [20–30.25]	25 [18–28]	22 [16–26.25]	19 [16–25.25]	**0.017**
SOFA score	10 [6.75–14]	7 [4–9]	8 [6–10]	6 [4–9]	**0.001**
Procalsitonin levels (ng/mL)	4.62 [1.12–24]	1.67 [0.3–8.3]	1.73 [0.54–6.3]	1.1 [0.28–4.5]	**0.047**
Calcium, ionized (mmol/L)	1.09 [1.0–1.2]	1.12 [1–1.23]	1.19 [1.05–1.25]	1.22 [1.12–1.33]	**0.001**
Albumin (g/dL)	2.3 [1.97–2.8]	2.7 [2.4–3.2]	2.8 [2.2–3.125]	2.8 [2.38–3.33]	**0.002**
Creatinine (mg/dL)	2.42 [1.32–3.8]	1.62 [0.84–3.23]	1.42 [0.8–3.28]	1.34 [0.7–2.75]	**0.019**
On admission					
Sepsis/septic shock	28 (56%)	15 (29.4%)	20 (40%)	9 (18%)	**0.001**
Renal dysfunction	26 (52%)	13 (21%)	16 (32%)	7 (14%)	**0.0001**
During ICU stay					
Invasive mechanical ventilation	29 (58%)	18 (35.3%)	16 (32%)	13 (26%)	**0.001**
Renal replacement therapy	25 (50%)	17 (33.3%)	17 (35.4%)	10 (20%)	**0.003**
ICU mortality	23 (46%)	19 (37.3%)	20 (40%)	14 (38%)	0.096

Values are presented as median [interquartile range] or *n* (%); APACHE: acute physiology and chronic health evaluation, SOFA: sequential organ failure assessment, and ICU: Intensive care unit.

**Table 4:** Characteristics of patients who survived (survivor) or died (nonsurvivor) in ICU.

Variable	Survivor (*n* = 125)	Nonsurvivor (*n* = 76)	*P* value
Age	66 [54–77]	66 [58–78]	0.755
Male gender	65 (52%)	48 (63%)	0.122
Length of hospital stay prior to ICU admission (days)	3 [2–6]	8 [2–20]	**0.001**
Length of ICU stay (days)	8 [5–12]	10 [6–20]	**0.008**
On admission			
APACHE II score	20 [16–25]	27 [23–31]	**0.0001**
SOFA score	6 [4–9]	10 [7–13]	**0.0001**
Glasgow coma scale	14 [13–15]	12 [8–15]	**0.0001**
Hemoglobin (g/dL)	10.4 [9.2–12.6]	9.5 [8.5–11]	**0.001**
Thrombocytes (/mm^3^)	176000 [114000–262000]	109000 [63750–173250]	**0.02**
C-reactive protein (mg/L)	81 [27–164]	125 [61–196]	**0.012**
Procalsitonin (ng/mL)	1.4 [0.3–6.2]	2.6 [1–10.4]	**0.008**
Calcium level (mg/dL)	8 [7.4–8.8]	7.6 [6.9–8.3]	**0.001**
Albumin level (g/dL)	2.9 [2.5–3.3]	2.3 [2–2.8]	**0.0001**
Oxygenation index	212 [144.5–282]	152 [94–233]	**0.0001**
Invasive mechanical ventilation	34 (27%)	54 (71%)	**0.0001**
Organ dysfunction	87 (70%)	73 (96%)	**0.0001**
Sepsis/septic shock	38 (30.4%)	34 (44.7%)	0.069
25 (OH) D < 20 ng/mL	79 (63%)	60 (79%)	**0.027**
Comorbidities			
Pulmonary diseases	43 (34%)	14 (18%)	**0.015**
Renal diseases	22 (18%)	24 (32%)	**0.022**
Malignancies	31 (25%)	30 (40%)	**0.028**
Admission from ER	63 (52%)	23 (30%)	**0.003**
During ICU			
Nosocomial infections	29 (23%)	42 (55%)	**0.0001**
Sepsis/septic shock	7 (5.6%)	59 (77.6%)	**0.0001**
Renal dysfunction	7 (6%)	23 (30%)	**0.0001**
Renal replacement therapy	26 (21%)	43 (57%)	**0.0001**
Mechanical ventilation	65 (52%)	63 (83%)	**0.0001**
Steroid requirement	55 (44%)	62 (83%)	**0.0001**
Invasive BP monitoring	47 (40%)	64 (89%)	**0.0001**
CVP monitoring	51 (43%)	68 (96%)	**0.0001**
Tracheostomy performed	6 (5%)	11 (16%)	**0.013**
Blood/blood product requirement	42 (35%)	53 (77%)	**0.0001**

Values are presented as median [interquartile range] or *n* (%); APACHE: acute physiology and chronic health evaluation, SOFA: sequential organ failure assessment, ICU: intensive care unit, BP: blood pressure, and CVP: central venous pressure.

**Table 5:** According to multivariate analysis, independent risk factors for ICU mortality*.

Variable	OR (95% CI)	*P* value
APACHE II score on admission	1.09 [1.02–1.17]	0.012
During ICU stay		
Mechanical ventilation	3.77 [1.13–12.55]	0.031
Renal dysfunction	5.22 [1.3–20.89]	0.02
Sepsis/septic shock	4.93 [1.69–14.34]	0.003
Steroid requirement	4.7 [1.6–13.62]	0.004
Blood/ blood product requirement	3.75 [1.4–9.95]	0.008

*Vit D insufficiency eliminated in second step of logistic regression analysis [*P* = 0.328; OR (95% CI): 1.8 (0.554–5.863].

## References

[1] Holick MF (2004). Vitamin D: importance in the prevention of cancers, type 1 diabetes, heart disease, and osteoporosis. *American Journal of Clinical Nutrition*.

[2] Adams JS, Hewison M (2010). Update in vitamin D. *Journal of Clinical Endocrinology and Metabolism*.

[3] Lee P, Eisman JA, Center JR (2009). Vitamin D deficiency in critically ill patients. *The New England Journal of Medicine*.

[4] Lee P (2011). How deficient are vitamin D deficient critically ill patients?. *Critical Care*.

[5] Lucidarme O, Messai E, Mazzoni T, Arcade M, Du Cheyron D (2010). Incidence and risk factors of vitamin D deficiency in critically ill patients: results from a prospective observational study. *Intensive Care Medicine*.

[6] Matthews LR, Ahmed Y, Wilson KL, Griggs DD, Danner OK (2012). Worsening severity of vitamin D deficiency is associated with increased length of stay, surgical intensive care unit cost, and mortality rate in surgical intensive care unit patients. *American Journal of Surgery*.

[7] Watkins RR, Yamshchikov AV, Lemonovich TL, Salata RA (2011). The role of vitamin D deficiency in sepsis and potential therapeutic implications. *Journal of Infection*.

[8] Henry HL, Bouillon R, Norman AW (2010). 14th Vitamin D Workshop consensus on vitamin D nutritional guidelines. *Journal of Steroid Biochemistry and Molecular Biology*.

[9] Krishnan A, Ochola J, Mundy J (2010). Acute fluid shifts influence the assessment of serum vitamin D status in critically ill patients. *Critical Care*.

[10] McKinney JD, Bailey BA, Garrett LH, Peiris P, Manning T, Peiris AN (2011). Relationship between vitamin D status and ICU outcomes in veterans. *Journal of the American Medical Directors Association*.

[11] Amrein K, Venkatesh B (2012). Vitamin D and the critically ill patient. *Current Opinion in Clinical Nutrition and Metabolic Care*.

[12] Baeke F, Takiishi T, Korf H, Gysemans C, Mathieu C (2010). Vitamin D: modulator of the immune system. *Current Opinion in Pharmacology*.

[13] Adams JS, Hewison M (2008). Unexpected actions of vitamin D: new perspectives on the regulation of innate and adaptive immunity. *Nature Clinical Practice Endocrinology and Metabolism*.

[14] Jeng L, Yamshchikov AV, Judd SE (2009). Alterations in vitamin D status and anti-microbial peptide levels in patients in the intensive care unit with sepsis. *Journal of Translational Medicine*.

[15] Amrein K, Sourij H, Wagner G (2011). Short-term effects of high-dose oral vitamin D3 in critically ill vitamin D deficient patients: a randomized, double-blind, placebo-controlled pilot study. *Critical Care*.

[16] Braun AB, Gibbons FK, Litonjua AA, Giovannucci E, Christopher KB (2012). Low serum 25-hydroxyvitamin D at critical care initiation is associated with increased mortality. *Critical Care Medicine*.

[17] Braun A, Chang D, Mahadevappa K (2011). Association of low serum 25-hydroxyvitamin D levels and mortality in the critically ill. *Critical Care Medicine*.

[18] Flynn L, Zimmerman LH, McNorton K (2012). Effects of vitamin D deficiency in critically ill surgical patients. *American Journal of Surgery*.

[19] Venkatram S, Chilimuri S, Adrish M, Salako A, Patel M, Diaz-Fuentes G (2011). Vitamin D deficiency is associated with mortality in the medical intensive care unit. *Critical Care*.

[20] van den Berghe G, van Roosbroeck D, Vanhove P, Wouters PJ, de Pourcq L, Bouillon R (2003). Bone turnover in prolonged critical illness: effect of vitamin D. *Journal of Clinical Endocrinology and Metabolism*.

[21] Arnson Y, Gringauz I, Itzhaky D (2012). Vitamin D deficiency is associated with poor outcomes and increased mortality in severely ill patients. *QJM*.

[22] Lee P (2011). Vitamin D metabolism and deficiency in critical illness. *Best Practice & Research Clinical Endocrinology & Metabolism*.

